# A Male with Cooccurrence of Down Syndrome and Fragile X Syndrome

**DOI:** 10.1155/2013/504695

**Published:** 2013-09-12

**Authors:** Tovi Anderson, Allison Buterbaugh, Kaitlin Love, Jeannie Visootsak

**Affiliations:** ^1^Emory University, Department of Human Genetics, 2165 N. Decatur Road, Decatur, GA 30033, USA; ^2^Florida State University College of Medicine, Tallahassee, FL 32304, USA

## Abstract

Down syndrome is the most common identifiable genetic cause of intellectual disability, with a unique physical gestalt that makes diagnosis possible during the newborn period. However, the physical characteristics of Fragile X syndrome are fairly subtle, resulting in the first clinical suspicion often arising from delayed developmental milestones. In addition, maladaptive behavior and autistic-like tendencies, such as hand flapping, poor eye contact, and hand biting, may be noted in Fragile X syndrome but are not as commonly observed in Down syndrome. Recognition of a potential secondary diagnosis, such as Fragile X syndrome, in individuals with Down syndrome is critical because there have been advances in targeted pharmacologic treatments for both conditions. Thus, an accurate diagnosis has implications in improving the individual's quality of life.

## 1. Introduction

Down syndrome (DS, OMIM #190685), or trisomy 21, is the most common genetic cause of intellectual disability. Brachycephaly with flat occiput, epicanthal folds and upslanting palpebral fissures, Brushfield spots in the iris, low nasal bridge, downturned mouth with protruding tongue, low-set ears, broad neck, and small hands with transverse crease are common features in DS [1]. Since the physical gestalt is straightforward, individuals with DS are typically diagnosed in the newborn period. 

The physical features of Fragile X syndrome (FXS, OMIM #300684) are often subtle; hence, clinical indication is based on an array of intellectual and emotional disabilities ranging from learning problems and autism to anxiety. Families typically become concerned about the child's development at an average age of 13 months, and a FXS diagnosis occurs at an average age of approximately 32 months [2]. The behavioral phenotype commonly associated with FXS may be helpful in suggesting the diagnosis. For instance, autistic-like features, such as hand flapping, hand biting, gaze avoidance, tactile defensiveness, and hyperarousal to sensory stimuli, are common in individuals with FXS [3, 4]. Additionally, anxiety, hyperactivity, impulsivity, and aggressive behavior can also be present [5]. 

The cooccurrence of DS and FXS in females has been reported in two cases prior to the identification of the *FMR1* gene in 1991 [7, 8]. Additionally, there has been one case report of a male with both DS and FXS [6]. Herein, we present the second case of a male with both DS and full mutation FXS, illustrating the importance of investigating additional diagnoses when a DS diagnosis does not appropriately explain the degree of intellectual disability and/or when there are other family members with developmental delay. Additionally, confirmation of a diagnosis of DS and/or FXS is critical, as a number of promising targeted treatments to improve neurodevelopmental and behavioral outcomes for both conditions are undergoing clinical trials. 

## 2. Clinical Report

**Figure 1:**
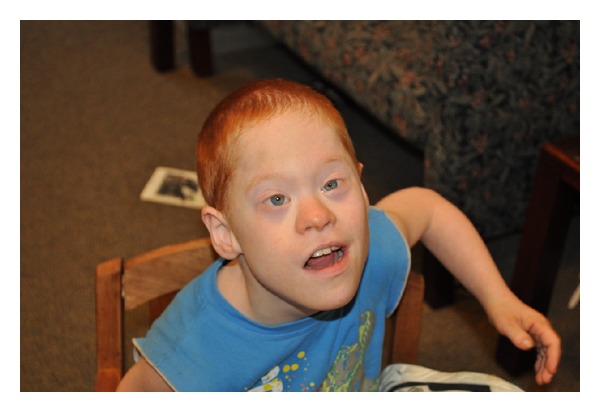
Boy with cooccurrence DS and full mutation FXS. Features include dolichocephaly, epicanthal folds, and upslanting palpebral fissures.

The patient was born at 41 weeks gestation by spontaneous vaginal delivery with a birth weight of 4.16 kg (>90th percentile), length of 51 cm (>90th percentile), and head circumference of 33.5 cm (25th–50th percentile). Pregnancy history was unremarkable. The clinical features of DS were recognized shortly after birth, including the following features; upslanting palpebral fissures, flat occiput, small nose, short neck with increased nuchal skin, clinodactyly of 5th fingers, short fingers, increased space between first and second toes bilaterally, and hypotonia (Figure 1). A karyotype confirmed the diagnosis of trisomy 21. The echocardiogram revealed a patent ductus arteriosus and ventricular septal defect. The patient was also diagnosed with Hirschsprung disease. 

Past medical history is remarkable for feeding difficulties, poor weight gain, hip dysplasia and neuromuscular hip dislocation, myringotomy for chronic otitis media, and adenoidectomy and tonsillectomy for chronic upper respiratory infections. Vision, hearing, and thyroid function tests have been normal. 

Developmental milestones were delayed, including sitting at 7 months and crawling at 8 months. He currently does not ambulate independently and continues to be hypotonic with ligamentous hyperlaxity. He is nonverbal and expresses his needs and wants with gestures. He is shy, affectionate, and has tactile defensiveness. He does not have mood instability, temper tantrums, anxiety, or stereotypic or self-injurious behaviors. He is currently 8 years and 11 months old and attends 4th grade special education classes for children with severe-profound intellectual disability. 

The patient is the only child born to his mother and father, at the ages of 24 years and 27 years, respectively. His parents are Caucasian and not known to be consanguineous. His mother has dyslexia and completed the 11th grade. The patient has three half-brothers (14, 12, and 2 years old) and one half-sister (16 months old) from his parents' previous relationships. They are developing appropriately for their age except for the patient's 2-year-old half-brother, who has developmental delay and maladaptive behavior, which prompted testing for FXS. Subsequently, he was diagnosed with FXS (270–1100 CGG repeats) with complete methylation. This diagnosis prompted testing for FXS in the patient at the age of 8 years and 8 months. The patient's results showed a full mutation of the *FMR1* gene (200–830 CGG repeats) with complete methylation. Both mutations of the *FMR1* gene were identified by PCR and Southern blot analysis. The mother has 120 CGG repeats based on PCR analysis. The patient's other half-siblings have not been tested for FXS. The patient's maternal grandmother is known to have attention deficit disorder, and the maternal grandfather is known to have dyslexia. The maternal grandmother reportedly has five nephews with FXS who are in their thirties.

On physical examination at the age of 8 years and 11 months, weight is 18.5 kg (<5th centile on the regular and DS growth charts), height is 102 cm (<5th centile on the regular and DS growth charts), and head circumference is 46 cm (<5th centile on the regular and DS growth charts). He is a thin boy with low tone and hyperextensible joints. He has typical features of DS, including dolichocephaly, epicanthal folds, upslanting palpebral fissures, and cutis laxa with prominent hand and foot creases. The right ear length is 5.5 cm (25th–50th percentile on a regular ear length chart; >50th percentile on a DS ear length chart) and left ear is 5.1 cm (10th percentile on a regular ear length chart; >50th percentile on a DS ear length chart).

Developmental outcomes are assessed at a chronological age of 8 years and 11 months using the *Vineland Adaptive Behavior Scales, Second Edition* [9]. The Vineland Scales assess personal and social sufficiency across three domains of functioning (mean score of 100 and standard deviation ± 15): Communication, Daily Living Skills, and Socialization. Results from the Vineland Scales reveal a Communication score of 43 (expressive language age equivalent of 6 months, receptive language age equivalent of 9 months), a Daily Living Skills score of 48, a Socialization score of 50, a Motor score of 40 (gross motor age equivalent of 8 months, fine motor age equivalent of 11 months), and an Adaptive Behavior score of 46. The motor score is the lowest, as the patient continues to have muscular hypotonia and ligamentous hyperlaxity and is unable to ambulate independently. 

The Child Behavior Checklist (CBCL) asks the parents to rate 112 problem behaviors on a three-point scale: (0) not true; (1) sometimes true; (2) often true. The CBCL consists of an internalizing domain (withdrawn, anxious/depressed, and somatic complaints), externalizing domain (aggressive behavior and delinquent behavior), and three other subdomains (social problems, thought problems, and attention problems) that sum for a total score [10]. The CBCL total domain raw scores are converted to *T* scores. Clinically significant *T* scores are those above 64, as established by Achenbach [10] using large epidemiological samples of children. This patient revealed an internalizing *T* score of 34, externalizing *T* score of 44, and total *T* score of 50, which indicates that he does not have maladaptive behavior, as the scores do not exceed the clinical cut-off score of 64.

## 3. Discussion

We present the second known case of a boy with both DS and full mutation FXS (Table 1). One other instance of a 14-year-old boy with both DS and full mutation FXS has been reported. His facial features are consistent with DS, but he also has a prominent forehead and macroorchidism which are physical characteristics of FXS. Additionally, he has severe hypotonia and joint laxity. However, our patient does not have physical characteristics associated with FXS, such as a long face and prominent ears [11]. The previously reported patient has global developmental delays, sat independently at 18 months, walked at 3 years, and at his current age is only intermittently using single words. He has Vineland Scale age equivalent of approximately 2 years and an adaptive composite score of 38, with strengths in daily living skills and weaknesses in communication. Unlike our reported case, this patient also has autism and severe behavioral issues including tantrums and physical aggression that have led to injury of others around him and necessitated his residence in a group home [6]. Of note, there are similarities between this case report and the patient we present, including both being born full-term at normal birth weights following uncomplicated pregnancies. Neither patient has severe heart defects requiring surgery, and both patients underwent myringotomies due to chronic otitis media. They also present with significant hypotonia, although the patient reported by Stevens et al. [6] is able to ambulate while our patient cannot. Also, both patients are extremely delayed in speech, with our patient being entirely nonverbal.

Two reported cases of females with cooccurrence of DS and FXS were described prior to the identification of the *FMR1* gene in 1991 [7, 8]. Collacott et al. [7] described a 21-year-old female of Afro-Caribbean origin with trisomy 21 and FXS with the Vineland Scales age equivalents of 21, 26, and 15 months in the Communication, Daily Living Skills, and Socialization domains, respectively. She is partially mobile, being unable to ambulate downstairs alternating her feet. Other maladaptive behaviors include hyperactivity, physical aggressiveness, and stereotypic behavior including rocking, biting her hands, and self-injurious behavior. Features of DS (e.g., epicanthal folds, upslanting palpebral fissures, brushfield spots, tongue protrusion) are noted. The case identified by Arinami et al. [8] is a 5-month-old Japanese girl with DS and FXS, who is severely hypotonic with physical features suggestive of FXS, including a long face with a prominent forehead, large ears, and a prominent jaw. In both cases, FXS testing was initiated because there were several family members with intellectual disability. 

Individuals with DS are typically diagnosed in the newborn period based on their physical features and hypotonia. However, the physical features of FXS are subtle; thus, clinical indication is based on intellectual and emotional disabilities and/or family history of FXS. Our patient was diagnosed with DS at birth based on his physical characteristics, and FXS was suspected after his younger half-brother was diagnosed with FXS. Additionally, he does not have the physical and behavioral features typically associated with FXS. However, his developmental profile is significantly delayed compared to other children with DS as evidenced by his inability to ambulate and communicate at the age of 8 years and 11 months. For these reasons, further clinical investigation should be considered in individuals with DS who appear to have developmental profiles significantly below what would be expected due to typical trisomy 21 and/or a family history of developmental delay. 

A correct diagnosis is becoming even more critical in patients with cooccurrence of DS and FXS due to the development of targeted pharmacological treatment options. Clinical trials for the treatment of FXS are ongoing and include a metabotropic glutamate receptor 5 (mGluR5) antagonist which has been shown to reverse the FXS phenotype in mice. Phase II and III clinical studies in FXS are presently underway for mGluR5 antagonists: R04917523 (Roche, Basel, Switzerland) and AFQ056 (Novartis, Basel, Switzerland). Targeting the gamma-aminobutyric acid (GABA) system may provide an alternative treatment strategy in FXS [12–14]. Studies are now being conducted to explore the efficacy, safety, and tolerability of arbaclofen, STX209, (Seaside Therapeutics, Boston, MA) in children and adults with FXS. Another FXS treatment has shown promise in clinical trials and acts to inhibit the activity of FMRP-regulated proteins through the use of the antibiotic minocycline [15, 16]. 

Targeted pharmacologic treatments for DS are also being developed, including a drug that acts as a GABA receptor antagonist (Hoffmann-La Roche, Basel, Switzerland) which may improve learning and memory in individuals with DS [17]. 

All four reported cases of individuals with a cooccurrence of DS and FXS were diagnosed with FXS based on a positive family history. However, even if a patient with DS is lacking a family history of FXS, testing for FXS should be considered if the patient presents with (1) more severe developmental delays and/or behavioral issues than the expected range for DS and/or (2) physical characteristics not consistent with a DS diagnosis. By following these guidelines, additional cases of dual diagnosis may be identified. Having a diagnosis of cooccurrence of DS and FXS will provide individually tailored treatments and resources, along with opportunities to participate in current pharmacologic treatment clinical trials that could maximize their potential. 

## Figures and Tables

**Table 1:** Cooccurrence of Down syndrome and Fragile X syndrome cases.

Case	Age	Sex	Medical problems	Physical characteristics		Development	Adaptive behavior composite score	Maladaptive behaviors
				DS	FXS			
Current case	8 y	M	PDA and VSD, Hirschsprung disease, hip dysplasia, myringotomy, adenoidectomy, tonsillectomy	+	−	Sitting at 7 months, does not ambulate independently, nonverbal	46	None

Stevens et al. (2010) [6]	14 y	M	ASD and mild aortic valve insufficiency, pressure equalization tubes, severe erosive esophagitis	+	+	Sitting at 18 months, ambulates independently, intermittent use of single words	38	Autism, tantrums, physical aggression

Collacott et al. (1990) [7]	21 y	F	None	+	+	Partially mobile, indistinct phrases	20	Stereotypic movements, hyperactivity, self-injurious behavior, physical aggression

Arinami et al. (1987) [8]	5 m	F	None	+	+	Unknown	Unknown	Unknown

PDA: patent ductus arteriosus.

VSD: ventricular septal defect.

ASD: atrial septal defect.
